# The role of DOT1L in the proliferation and prognosis of gastric cancer

**DOI:** 10.1042/BSR20193515_RET

**Published:** 2026-04-10

**Authors:** Jun Qian, Zaozhi Song, Zhuoli Wei, Qingkang Wang, Xinxin Zhang, Xiaoying Tao, Nan Wu, Xue Liu

**Affiliations:** Department of Surgical Oncology, The First Affiliat-ed Hospital of Bengbu Medical College, Bengbu, China

**Keywords:** DOT1L, EPZ5676, Gastric cancer, Prognosis

This article is being retracted from *Bioscience Reports* at the request of the Editor-in-Chief and the Editorial Board. This follows the receipt of a notification from a reader, alerting the Editorial Board to similarities within the manuscript, as well as concerns over the experimental design and ethics statement.

Specifically, these include the following concerns:

- In Figure 3, there are similar markings that effect multiple bands
- Similarities have been identified in Figure 3A between the first two SGC7901/CDK4 bands and the last two MGC803/CDK4 bands.
- Within the experimental design, the methods state that EPZ5676 was administered weekly, however in the Figure 4 legend it is stated that EPZ5676 administration occurs twice weekly.
- Reference is made to the Ethics Committee of the Institute, without detailing the specific Institution.

The authors have been contacted with regards to the retraction and have not responded to the Journal's queries or the concerns raised. Given the extent of the issues raised, the Editorial Board stands by the decision to retract the article.

